# Triplet Exciton Sensitization of Silicon Mediated by Defect States in Hafnium Oxynitride

**DOI:** 10.1002/adma.202415110

**Published:** 2024-12-23

**Authors:** Narumi Nagaya, Alexandra Alexiu, Collin F. Perkinson, Oliver M. Nix, Dooyong Koh, Moungi G. Bawendi, William A. Tisdale, Troy Van Voorhis, Marc A. Baldo

**Affiliations:** ^1^ Research Laboratory of Electronics Massachusetts Institute of Technology 77 Massachusetts Avenue Cambridge MA 02139 USA; ^2^ Department of Chemical Engineering Massachusetts Institute of Technology 77 Massachusetts Avenue Cambridge MA 02139 USA; ^3^ Department of Chemistry Massachusetts Institute of Technology 77 Massachusetts Avenue Cambridge MA 02139 USA

**Keywords:** DFT calculations, photovoltaics, singlet fission, triplet exciton sensitization

## Abstract

Singlet exciton fission has the potential to increase the efficiency of crystalline silicon solar cells beyond the conventional single junction limit. Perhaps the largest obstacle to achieving this enhancement is uncertainty about energy coupling mechanisms at the interfaces between silicon and exciton fission materials such as tetracene. Here, the previously reported silicon‐hafnium oxynitride‐tetracene structure is studied and a combination of magnetic‐field‐dependent silicon photoluminescence measurements and density functional theory calculations is used to probe the influence of the interlayer composition on the triplet transfer process across the hafnium oxynitride interlayer. It is found that hafnium oxide interlayers do not show triplet exciton sensitization of silicon, and that nitrogen content in hafnium oxynitride layers is correlated with enhanced sensitization. Calculation results reveal that defects in hafnium oxynitride interlayers with higher nitrogen content introduce states close to the band‐edge of silicon, which can mediate the triplet exciton transfer process. Some defects introduce additional deleterious mid‐gap states, which may explain observed silicon photoluminescence quenching. These results show that band‐edge states can mediate the triplet exciton transfer process, potentially through a sequential charge transfer mechanism.

## Graphical abstract

This work investigates the influence of hafnium oxynitride interlayer composition on triplet exciton sensitization of singlet fission solar cells. Nitrogen content in hafnium oxynitride layers is correlated with enhanced sensitization. Calculation results reveal that defects in hafnium oxynitride interlayers with higher nitrogen content introduce states close to the band‐edge of silicon, which can mediate the triplet exciton transfer process.

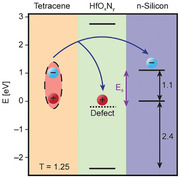

## 1 Introduction

The adoption of crystalline silicon (*c*‐Si) photovoltaics is limited by the price of solar cells and the cost of their installation. Improving cell efficiency is an important goal because maximizing energy generation reduces the effective cost of both cells and installation. Singlet exciton fission has been proposed as a method for enhancing Si solar cell efficiencies beyond the conventional theoretical limit for single junction devices.^[^ ^1^ ^]^ It generates two triplet excitons from one singlet exciton.^[^ ^2^ ^]^ If a singlet fission material such as tetracene (Tc) is used to absorb the high energy photons of the solar spectrum, then transfer of the resulting triplet excitons to *c*‐Si could increase Si cell efficiencies from 29% to 35%–42%.^[^ ^3^, ^4^, ^5^ ^]^

Unfortunately, the transfer of triplet excitons directly from Tc to *c*‐Si has proven to be exceptionally challenging.^[^ ^6^, ^7^, ^8^, ^9^, ^10^ ^]^ The fundamental obstacle is that Tc triplets are non‐emissive states and incapable of near‐field or radiative coupling to *c*‐Si. Instead, triplet diffusion in molecular films typically relies on Dexter transport and involves simultaneous tunneling of the electron and hole from donor to acceptor molecules. Tunneling is inherently short range, limiting the thickness of Si passivation layers, and increasing the impact of Si surface defect states that quench triplet excitons.^[^ ^11^ ^]^

**Figure 1:**
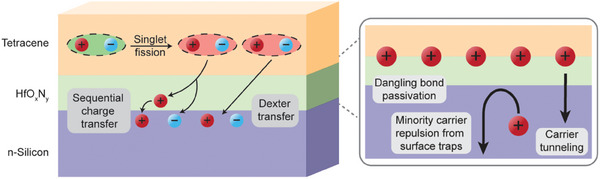
Schematic of the n‐silicon‐HfO_x_N_y_‐tetracene structure studied in this work. Triplet excitons are formed from the singlet excitons in the Tc layer through singlet fission. The triplet excitons can then either undergo a sequential charge transfer process or a Dexter transfer process to transfer to the n‐Si layer. The HfO_x_N_y_ interlayer provides chemical passivation by reacting with dangling bonds on the Si surface. It also provides electric field‐effect passivation of the Si surface such that minority carriers in n‐Si are repelled from surface trap states. This passivation is proposed to be mediated by defect states.^[^ ^11^ ^]^ The optimum interlayer thickness is determined by the interplay between tunneling distance of the transferred carriers and Si surface passivation.

Previous bichromatic magnetic field‐dependent measurements show that using a thin layer of hafnium oxynitride (HfO_x_N_y_) between Tc and n‐type *c*‐Si (n‐Si) enables triplet exciton sensitization of *c*‐Si.^[^ ^11^ ^]^ The sensitization effect is strongly dependent on the thickness of the HfO_x_N_y_ interlayer, with an optimum thickness of 8 Å, attributed to the interplay between carrier tunneling distance and Si surface passivation. Both passivation and energy transfer processes are schematically summarized in **Figure** **1**. HfO_x_N_y_ is expected to provide chemical passivation of dangling bonds at the Si surface. The Si‐HfO_x_N_y_‐Tc samples also exhibit electric field‐effect passivation when optically exciting both Tc and/or n‐Si.^[^ ^11^ ^]^ Trapping of minority (hole) carriers in the HfO_x_N_y_ interlayer is thought to be largely responsible for the electric field passivation effect. The trapped positive charge repels minority carriers from the surface of n‐Si, reducing the surface recombination velocity. In contrast, electron traps only slightly affect the concentration of the majority carriers and have little effect on the rate of surface recombination.

Two potential mechanisms have been proposed to explain triplet exciton transfer from Tc to *c*‐Si through a HfO_x_N_y_ layer.^[^ ^11^ ^]^ As depicted in Figure 1, the triplet exciton could transfer through a sequential charge transfer mechanism, where the triplet exciton dissociates and the electron and hole transfer successively one after the other,^[^ ^5^ ^]^ or through a Dexter transfer mechanism,^[^ ^12^ ^]^ where the electron and hole transfer simultaneously to the Si. Previous studies of LiF interlayers,^[^ ^6^, ^7^ ^]^ pyrene passivation layers^[^ ^8^ ^]^ and covalently bound tetracene‐derivative seed layers,^[^ ^10^ ^]^ have not provided strong support for the effectiveness of Dexter transport at interfaces between molecules and *c*‐Si.

The measured band alignment with the HfO_x_N_y_ interlayer^[^ ^11^ ^]^ also does not appear to support the sequential charge transfer mechanism of the triplet excitons. The previous observation of electric field‐effect passivation,^[^ ^11^ ^]^ however, points to the presence of defects in the HfO_x_N_y_ interlayer. In this work, we explore the potential role these defect states could play in the triplet exciton sensitization process by varying the composition of HfO_x_N_y_ interlayers and fabricating optical Si‐HfO_x_N_y_‐Tc samples to measure the interlayer‐thickness‐dependent Si photoluminescence (PL) change of these samples under an external magnetic field. We correlate the experimental observations to density functional theory (DFT) calculations of defect state positions in these interlayers. Our results suggest that defect states in HfO_x_N_y_ are mediating sequential charge transfer of the triplet exciton.

## 2 Results and Discussion

HfO_x_N_y_ is a complex, non‐stoichiometric material. To explore its properties and function at the interface between Si and Tc, we compare different compositions of HfO_x_N_y_. The limiting case of hafnium oxide (HfO_x_) is particularly important because HfO_x_ has been shown to be a good passivating interlayer in Si solar cells, due to both chemical passivation of Si dangling bonds and field‐effect passivation.^[^ ^13^, ^14^, ^15^ ^]^ HfO_x_ thin films can either be positively or negatively doped, depending on their stoichiometry.^[^ ^15^ ^]^ In materials with a large concentration of oxygen vacancies, HfO_x_ films develop a build‐up of positive charges which electrostatically passivate n‐doped Si.^[^ ^13^ ^]^

### 2.1 HfO_x_N_y_ Composition Variation

**Figure 2:**
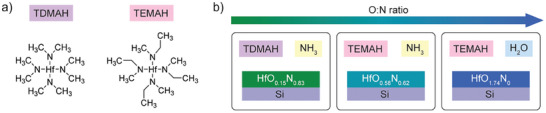
a) Hafnium precursors used to grow the HfO_x_N_y_ interlayers in this work: tetrakis(dimethylamino)hafnium (TDMAH) and tetrakis(ethylmethylamino)hafnium (TEMAH). b) Combinations of precursors used in the atomic layer deposition process for each layer and the corresponding compositions of the HfO_x_N_y_ films that were grown. The compositions in the schematic were obtained using X‐ray photoelectron spectroscopy depth profiling by in situ sputtering on thicker films and selecting the compositions measured at the depth point just before the Si substrate.

We grow HfO_x_N_y_ films on cleaned Si surfaces using atomic layer deposition (ALD) with different precursors to achieve varying ratios of O:N. **Figure** **2**a shows the chemical structures of the two hafnium precursors used in this work: tetrakis(dimethylamino)hafnium (TDMAH) and tetrakis(ethylmethylamino)hafnium (TEMAH). The HfO_x_N_y_ interlayers are grown following recipes for growth of Hf_3_N_4_.^[^ ^11^, ^16^ ^]^ The oxygen present in the films could come from residual oxygen in the reaction chamber, as well as from oxygen diffusion through post‐deposition exposure of the films to air. TEMAH is reported to show unfavorable reactivity with the nitrogen precursor ammonia (NH_3_).^[^ ^16^ ^]^ Thus, we expect the HfO_x_N_y_ films grown using TEMAH to have a lower nitrogen content. We also prepare a HfO_x_ film for comparison replacing the nitrogen precursor with an oxygen precursor. To evaluate the chemical compositions of the HfO_x_N_y_ films, we use X‐ray photoelectron spectroscopy (XPS). Because sensitization of Si requires ultrathin oxynitride films,^[^ ^11^ ^]^ the measured compositions have a strong Si and oxygen background from the substrate, as well as carbon contamination on the surface due to film exposure to the atmosphere. To make comparisons of the compositions across the samples, we prepare thicker films and sputter them to obtain composition depth profiles (see Figures S1–S3, Supporting Information). For convenience, we henceforth refer to the films by their measured compositions just before Si is detected in the depth profile (i.e. film composition close to the silicon surface, which is, ideally, representative of the thin films grown on silicon). The precursor combinations and aforementioned compositions of the corresponding HfO_x_N_y_ films are presented in Figure 2b.

### 2.2 Optical Sample Characterization

To investigate the triplet sensitization efficiencies, we prepare optical samples of Si with the deposited HfO_x_N_y_ interlayers of different thicknesses, and we then deposit 30 nm of Tc on top. We characterize the optical samples by measuring the Si PL change under an applied magnetic field, as previously conducted by Einzinger et al.^[^ ^11^ ^]^

**Figure 3:**
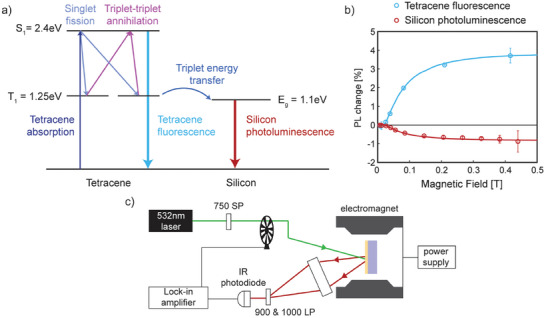
a) Schematic showing the processes that can occur in the Si‐HfO_x_N_y_‐Tc optical sample. Tc can absorb photons to generate singlet excitons that can undergo singlet fission to form two triplet excitons each. The triplet excitons could either combine via triplet‐triplet annihilation to form a singlet exciton or transfer to the Si. Tc singlet excitons can decay to the ground state to emit fluorescence. The excitedSi can decay to the ground state to emit photoluminescence. b) PL change measured as a function of magnetic field for Tc fluorescence and for Si PL. The solid lines are fits to the singlet fission characteristic curve in Tc. c) The experimental setup used to measure the Si PL change as a function of the magnetic field. See Methods for full details.

When the Tc layer is excited, it absorbs photons to generate singlet excitons. The singlet exciton can undergo singlet fission to generate a correlated triplet pair state with spin‐singlet character, which either diffuses into separated triplet excitons or recombines through triplet‐triplet annihilation to form a singlet exciton. Triplet excitons in Tc can also transfer their energy to Si. **Figure** **3**a shows a summary of the processes that can occur in the Si‐HfO_x_N_y_‐Tc optical samples. The coupling of the singlet exciton and the correlated triplet pair state can be modulated by a magnetic field, affecting the equilibrium population of singlet and triplet excitons, as described by the Merrifield model.^[^ ^17^ ^]^ At low fields, the overall singlet fission rate is increased, followed by a decrease at high fields. This results in a characteristic response of the steady‐state Tc fluorescence signal to an external magnetic field, as shown in Figure 3b.^[^ ^18^ ^]^ Measuring the Si PL shows the same characteristic response but inverted, which aligns with the expectation that triplet excitons from Tc are sensitizing the Si. It should be noted that the sensitization effects potentially include both energy transfer and electric field‐effect passivation induced by defect charging at the interface.^[^ ^11^ ^]^

To infer the efficiency of triplet exciton sensitization of Si, we measure the percentage change in Si PL from the Si‐HfO_x_N_y_‐Tc samples upon application of a 0.4 T magnetic field. The samples are excited by a 532 nm laser source and the Si PL is captured and focused onto an IR photodetector. The experimental setup is depicted in Figure 3c.

**Figure 4:**
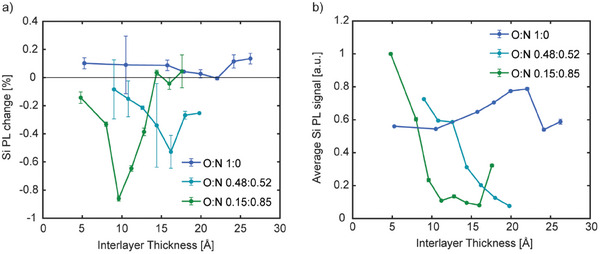
a) Percentage Si PL change of Si‐HfO_x_N_y_‐Tc samples upon application of a 0.4 T magnetic field at different HfO_x_N_y_ interlayer thicknesses and compositions. The magnitude of the Si PL change increases with higher nitrogen content in the HfO_x_N_y_ layer. b) Average Si PL signal of Si‐HfO_x_N_y_‐Tc samples at different HfO_x_N_y_ interlayer thicknesses and compositions. The average Si PL is roughly constant with thickness for HfO_x_, but trends downward with increasing thickness of the HfO_x_N_y_ interlayers.

Previous work on HfO_x_N_y_ layers found a strong thickness‐dependence of the sensitization effect.^[^ ^11^ ^]^ As a result, we measure the interlayer thickness‐dependent Si PL change for the samples with different interlayer compositions. The results are presented in **Figure** **4**a). Notably, changing the interlayer composition affects both the magnitude of the Si PL change and the thickness‐dependence. The HfO_x_ films with no nitrogen showed no negative Si PL change with an applied field at all thicknesses, implying no triplet exciton sensitization of the Si. With increasing nitrogen content in the HfO_x_N_y_ interlayer, the samples exhibit a higher magnitude of Si PL change with an applied magnetic field, implying a greater sensitization effect. The optimum thickness also appears to decrease with greater nitrogen content.

We also measure the average Si PL signal as a function of interlayer thickness and composition (Figure 4b). We again observe differences in the trends between samples with the HfO_x_ and HfO_x_N_y_ interlayers. Unlike the thickness‐dependence of the samples with the HfO_x_ interlayers, the samples with both HfO_x_N_y_ interlayers show a nearly monotonically decreasing Si PL signal with increasing interlayer thickness.

The quenching of Si PL in the HfO_x_N_y_ interlayers is an indicator of mid‐ and near‐band‐edge defect states. In combination with observed enhanced triplet sensitization, we propose that triplet excitons are transferred through a sequential charge transfer mechanism, mediated by defect states in the HfO_x_N_y_ layer.

### 2.3 Computational Investigation of HfO_x_N_y_

To evaluate the hypothesis that defect states near the band‐edge of Si may support sequential charge transfer of the triplet excitons, we perform DFT calculations to assess the density of states (DOS) for a range of materials of interest.

We start with stoichiometric hafnium oxide (HfO_2_), a well‐studied wide band gap metal oxide. Given that polycrystalline phases only appear after annealing at high temperatures (>500 °C),^[^ ^19^, ^20^ ^]^ most hafnia thin films reported in the literature exhibit amorphous structures. A lack of crystallinity precludes us from making direct comparisons with experiments. Therefore, we choose to focus on the thermally accessible phases of HfO_2_, which are the monoclinic ground state (*P2_1_/c*) and the orthorhombic state (*Pbca*), which is 11.1 meV/atom higher in energy (see Figure S6, Supporting Information for energy diagram). A brief analysis of point defects in monoclinic HfO_2_ is reported in Figure S7 (Supporting Information), which agrees with previous computational and experimental results.^[^ ^21^, ^22^, ^23^ ^]^ Overwhelmingly, these studies show that O vacancies in HfO_2_ can act as electron traps, and defects associated with N atoms may cause additional trap states. Note that our calculations employ the global hybrid PBE0,^[^ ^24^, ^25^ ^]^ which dramatically improves semiconductor band gap and level alignment compared to the generalized gradient approximation (GGA) used in earlier work. However, at least in the case of HfO_2_, the qualitative conclusions are unchanged.

To extend the investigation to HfO_x_N_y_, we carry out a systematic interpolation between HfO_2_ and Hf_3_N_4_, to establish the likelihood that different O:N ratios will be conducive to efficient triplet exciton transfer from Tc to Si. For a smooth interpolation, the two endmembers should have similar crystal structures, which leads us to focus on orthorhombic HfO_2_ (*Pbca*) and Hf_3_N_4_ (*Pnma*), as there is no thermally accessible monoclinic Hf_3_N_4_.

**Figure 5:**
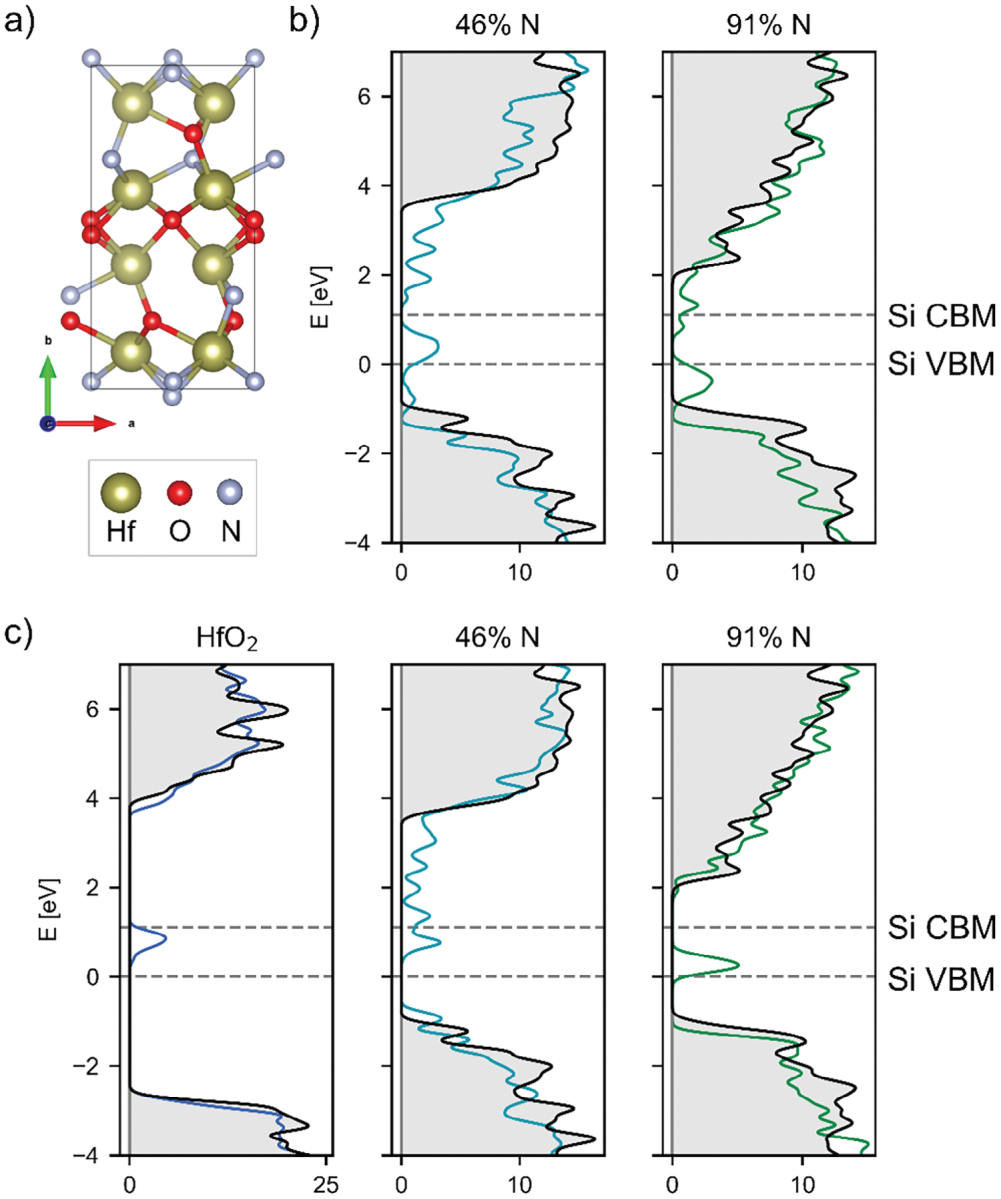
a) Representative unit cell of HfO_x_N_y_ (with 46% N). b) Density of states plots for N vacancy defects in two representative HfO_x_N_y_ compositions. The DOS of the pristine unit cell (grey shaded region) is compared with the defective DOS (colored). The Si valence band maximum (VBM) and conduction band minimum (CBM) are marked by dotted grey lines. The energy axis is shifted such that the Si VBM is at 0 energy. c) DOS plots for O vacancy defects in HfO_2_ and HfO_x_N_y_.

Oxygen atoms were progressively substituted by nitrogen atoms, maintaining the overall stoichiometry, to yield a range of compositions of the form HfO_x_N_y_, with N substitution percentages varying from 13–91% (see **Figure** **5**a for a representative unit cell). The Si band gap and offset between the HfO_x_N_y_ and Si valence band‐edges are assumed to be 1.1 and 2.4 eV, respectively, as calculated from ultraviolet photoelectron spectroscopy experiments by Einzinger et al.^[^ ^11^ ^]^ Other experimental works obtain a range of values between 2.4–3 eV^[^ ^26^, ^27^, ^28^, ^29^, ^30^ ^]^ for the valence band offset, depending on the exact deposition setup and any post‐deposition annealing treatments. We highlight that our computational results will be somewhat dependent on this exact value, however we choose 2.4 eV as the valence band offset for consistency with the experiments of Einzinger et al.^[^ ^11^ ^]^

In agreement with previous experimental and computational results,^[^ ^31^, ^32^, ^33^ ^]^ the band gap of HfO_x_N_y_ decreases monotonically with increasing N content (see Figure S5, Supporting Information). The band gaps of all pristine samples are free of any energy states that could realistically mediate sequential charge transfer of triplet excitons to Si.

#### 2.3.1 Defective Structures

We introduced a variety of point defects: O, N, and Hf vacancies. All defects considered are charge neutral, to remove the dependence of their formation energy on the Fermi level. An analysis of charged defects could make the object of a further study. Hf vacancies form no mid‐gap states of interest, so we reserve their discussion for Figure S10 (Supporting Information).

Representative DOS plots are shown in Figure 5b for N vacancies in two HfO_x_N_y_ compositions, a more N poor case (46% N) that exhibits a trap state in the middle of the Si band gap and a N rich case (91% N) where the defect state could conceivably trap holes. Similarly for O vacancies, a comparison is shown between the same two HfO_x_N_y_ compositions and HfO_2_ in Figure 5c.

**Figure 6:**
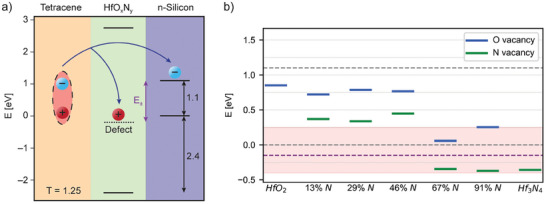
a) Schematic showing the energy level alignment of the Si‐HfO_x_N_y_‐Tc structure, as previously reported.^[^ ^11^ ^]^ Overlaid, one possible example of a defect state in HfO_x_N_y_ helping mediate the triplet exciton dissociation and sequential charge transfer process of the triplet exciton from Tc. This forms an intermediate charge‐separated state with energy E_±_ (assuming negligible binding energy^[^ ^34^, ^35^ ^]^), where the electron is on the Si conduction band‐edge and the hole is on the defect state level in HfO_x_N_y_. b) Summary of defect energy levels caused by O vacancies (blue) and N vacancies (green). The Si valence band maximum (VBM) and conduction band minimum (CBM) are marked with dotted grey lines, and the dotted purple line represents the minimum energy a defect state could have such that *E*_±_ ≤ *E*_*T*,*Tc*_. The red shaded box marks the energy region for potential hole traps. The energy axis is shifted such that the Si VBM is at 0 energy.

One possible pathway for sequential charge transfer mediated by a defect state in HfO_x_N_y_ is presented in **Figure** **6**a, where the electron is initially transferred to the Si conduction band (E_C_) and the hole is supported at a HfO_x_N_y_ defect state (E_D_) near the valence band‐edge of Si. The hole is then subsequently transferred to the Si valence band (E_V_).

Such a defect state needs to have significant density of states close to the Si valence band‐edge, and ideally, the energy of the charge‐separated state (*E*_±_) should be lower than the triplet energy in Tc (≈1.25 eV, within thermal energy)^[^ ^11^ ^]^: *E*_±_ ≤ *E*_*T*,*Tc*_. We assume binding energy can be neglected for the charge‐separated state energy due to expected charge screening in Si.^[^ ^34^, ^35^ ^]^ Furthermore, the energy barrier for the subsequent hole transfer from HfO_x_N_y_ to Si must be sufficiently low, on the order of thermal energy at room temperature, to avoid permanent trapping of the minority carriers in defect states.

A summary of all vacancy energy levels is shown in Figure 6b, for HfO_2_, HfO_x_N_y_, and Hf_3_N_4_. Only the maximum of each defect peak in the DOS plots is shown, although each peak is in reality broadened by up to 0.5 eV each (see Figure 5b,c for examples of realistic DOS plots, as well as Figures S8–S12 (Supporting Information) for DOS plots of all possible defects).

O vacancies cause electron traps for low N compositions, shifting to potential hole traps as the N content increases. N vacancies generally lead to the formation of hole traps; for higher N content, the defect states can even lie slightly below the Si VBM, which could promote a barrierless hole transfer from the defect energy level to the Si VBM (see Figure 6a). The overall trend is for vacancy defect levels to decrease in energy as the N content increases, with the optimal composition for hole trap formation being higher than 67% N.

#### 2.3.2 Defect Formation Energies

The relative alignment of potential trap states offers an explanation for which types of defects could be responsible for the success of using the HfO_x_N_y_ interlayer in singlet fission solar cells, but gives no information about the prevalence of such defects. Computing their formation energies provides some insight into their relative thermodynamic stabilities and the likelihood of defect formation in a real material.

**Figure 7:**
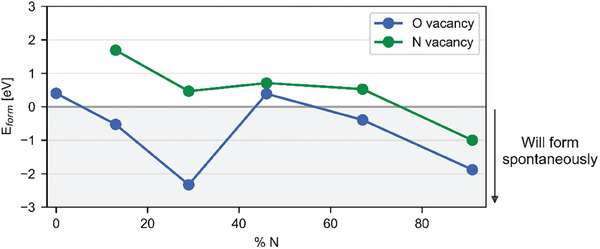
Formation energies for O vacancies (blue) and N vacancies (green), for HfO_2_ and HfO_x_N_y_ compositions. O vacancies form more readily than N vacancies, with both forming spontaneously at the 91% N composition.

Defect formation energies and concentrations are highly dependent on the conditions under which these materials are synthesized, most notably the experimental temperature and the chemical potentials, µ_N_ and µ_O_, of N and O. Making reasonable approximations to µ_N_ and μ_O_ (see computational details in Methods) leads to the formation energies shown in **Figure** **7**. We see that for all O:N ratios, oxygen vacancies are more stable than nitrogen vacancies, which would in turn indicate that we should expect oxygen vacancies to be more common. As implied by the low or even negative formation energies, oxygen vacancies form readily at any composition (negative formation energies suggest spontaneous defect formation). The second observation is that nitrogen vacancies are systematically more stable for higher nitrogen composition as compared to lower, suggesting that more nitrogen‐rich HfO_x_N_y_ should have more nitrogen defects.

Using a temperature of 423 K, in line with the reaction chamber temperature used in our experimental work, and assuming the structure is thermodynamically (rather than kinetically) determined, we can also estimate defect concentrations using an Arrhenius‐like expression (Table S5, Supporting Information). Oxygen vacancy concentrations are large, irrespective of the HfO_x_N_y_ composition, estimated to be on the order of 10^16^ cm^−3^ in HfO_2_. This estimate is in accordance with previous computational^[^ ^36^ ^]^ and experimental^[^ ^37^, ^38^ ^]^ studies, and may even be underestimating the concentration of oxygen vacancies in a real material. The highest nitrogen vacancy concentrations are obtained for the material with 91% N, which has a negative formation energy that suggests spontaneous defect formation. These quantitative predictions confirm the qualitative expectations implied by the defect‐induced energy levels in Figure 6b.

This computational analysis suggests that HfO_x_N_y_ with higher N content supports the formation of defect states near the valence band‐)edge of Si, helping to mediate the sequential charge transfer mechanism proposed in Figure 6a. This hypothesis is consistent with the experimentally observed lower optimum transfer thickness and higher Si PL change magnitude for HfO_x_N_y_ films with higher N concentration. Additionally, the defect formation energy calculations suggest that HfO_x_N_y_ films with higher N concentrations could favor the formation of N vacancies compared to the films with moderate N concentrations.

For the HfO_x_ layer, calculations suggest that oxygen vacancies could result in defect states near the conduction band of Si, which could support triplet exciton transfer through an initial hole transfer. However, we note that the magnetic field‐dependent measurements detect both energy transfer and passivation. The wafers used in the study are n‐doped with holes as the minority carriers. Thus, the electrons in the HfO_x_ layer create an electric field that attracts the minority carriers to the surface, resulting in recombination of any transferred triplet excitons.

Our calculations also support the observation of nearly monotonically decreasing Si PL signal with increasing HfO_x_N_y_ interlayer thickness, consistent with the increased number of mid‐gap defect‐induced states in thicker interlayers from nitrogen and oxygen. The defect formation studies for the HfO_x_ layer also suggest the presence of fewer mid‐gap defect states, consistent with the experimentally‐observed relatively constant Si PL signal with thickness.

## 3 Conclusion

In conclusion, we show that sequential charge transfer of triplet excitons from Tc to *c*‐Si can be mediated through defect states in the HfO_x_N_y_ interlayer. Our results suggest that HfO_x_N_y_ films with higher nitrogen content are more likely to form N vacancy defects, introducing a density of near‐band‐edge states that lie below the Si VBM. These states correlate with increased magnitudes of magnetic field‐induced modulation of Si PL and reduced optimum interlayer thickness for triplet sensitization of Si. We also find, however, that defects in HfO_x_N_y_ interlayers introduce many deep mid‐gap states which can quench the overall Si PL. Future efforts for achieving high efficiency singlet fission‐sensitized Si photovoltaics will require interlayers that account for the importance of both near‐band‐edge states and electric field passivation of the Si surface.

## 4 Experimental Section

### Materials

n‐doped prime‐grade single‐side‐polished silicon wafers with 0.25–0.75 Ωcm resistivity were purchased from UniversityWafer. Tetracene was purchased from Sigma–Aldrich (99.99% purity) and purified via sublimation in a tube furnace once.

### Sample Preparation

The silicon wafers were diced into 1‐inch squares and cleaned by sonicating in detergent solution (Micro‐90), deionized water, and acetone, followed by immersion in boiling isopropanol. The wafers were then dried with pressurized nitrogen and transferred to a clean room. A standard Radio Corporation of America (RCA) cleaning process on the wafers was performed, which left a thin oxide layer on the silicon surface. Following the clean, the wafers were quickly transferred for atomic layer deposition (ALD) of the HfO_x_N_y_ layers (Cambridge Nanotech Savannah). The base pressure of the chamber was 0.4 torr at a nitrogen flow of 40 cm^3^ min^−1^.

For the HfO_0.15_N_0.83_ layer, tetrakis(dimethylamino)hafnium (TDMAH) was used as the Hf precursor and ammonia was used as the nitrogen precursor, with oxygen likely from residual oxygen in the chamber and from film exposure to air post‐deposition. The TDMAH precursor was heated to 75 °C and the ALD reaction chamber was set to 150 °C. The TDMAH precursor was pulsed for 30 ms, and the NH_3_ precursor was pulsed for 15 ms for every cycle.

For the HfO_0.58_N_0.62_ layer, tetrakis(ethylmethylamino)hafnium (TEMAH) was used as the Hf precursor and NH_3_ was used as the nitrogen precursor, with oxygen likely from residual oxygen in the chamber and from film exposure to air post‐deposition. The TEMAH precursor was heated to 110 °C and the ALD reaction chamber was set to 150 °C. The TEMAH precursor was pulsed for 400 ms, and the NH_3_ precursor was pulsed for 15 ms for every cycle.

For the HfO_1.74_ layer, TEMAH was used as the Hf precursor and water was used as the oxygen precursor. The TEMAH precursor was heated to 110 °C and the ALD reaction chamber was set to 200 °C. The TEMAH precursor was pulsed for 250 ms, and the H_2_O precursor was pulsed for 15 ms for every cycle.

After deposition of the HfO_x_N_y_ interlayer, the samples were quickly transferred to a vacuum chamber at a pressure of <  ×10^−6^ torr for tetracene deposition via thermal evaporation. 30 nm of tetracene was evaporated at a rate of 1 Å s^−1^. The samples were then encapsulated with a glass slide and ultraviolet curable epoxy in a dry nitrogen atmosphere (<1 ppm O_2_). A square foil piece was used to cover the active area during UV exposure.

### X‐ray Photoelectron Spectroscopy (XPS)

XPS measurements were performed using a PHI VersaProbe II X‐ray photoelectron spectrometer with monochromated Al K‐α X‐rays. Compositional depth‐profiles were obtained by sputtering the surface with C_60_ ions (operated at 2 kV, 1 µA, over a 2 × 2‐mm area) and measuring the photoelectron peak areas for C1s, N1s, O1s, Si2p, Hf4f in 1‐min intervals. The reported compositions were obtained after sputtering on 250‐cycle films and selecting a depth profile point close to the silicon wafer surface to remove the effects of surface impurities and present compositions that were close to the sample surface.

### Magnetic‐Field‐Dependent Photoluminescence Measurements

Optical samples were placed in Voigt geometry (perpendicular to the magnetic field) between the poles of an electromagnet and excited by a mechanically‐chopped (281 Hz) 200 mW 532‐nm laser (Coherent Verdi G18). A 750 nm shortpass filter was placed in the excitation path to ensure only 532 nm light would excite the sample. The tetracene fluorescence was collected by a silicon photodetector (Newport 818‐SL) connected to a lock‐in amplifier (Stanford Research Systems SR 830), with a 532 nm notch filter and a 550 nm longpass filter in front of the detector. The two filters were used to block out the laser contribution to the signal detected by the photodetector. The silicon photoluminescence was collected by an IR photodetector (Newport 818‐IR), with 900 and 1000 nm longpass filters in front of the detector to ensure collection of only the silicon photoluminescence which tails off at ≈800 nm (see Figure S4, Supporting Information). The electromagnet was periodically switched on and off at different field strengths for four cycle loops at each field strength to obtain the measurements in Figure 6b. For the thickness‐dependent magnetic‐field‐dependent measurements, the above experiments were conducted for a magnetic field strength of 0.4 T, as measured by a gaussmeter (Lakeshore HMMT‐6J04‐VF).

### Computational Methods

All calculations were performed using density functional theory at the hybrid functional level (PBE0), using the “light” default settings for basis sets and numerical integration grid settings, as defined in the FHI‐aims software.^[^ ^39^, ^40^, ^41^, ^42^ ^]^ Following convergence tests (see Figure S9, Supporting Information), a 2 × 2 × 2 k‐point grid was chosen for the geometry optimizations and a 4 × 4 × 4 grid for the density of states calculations. For a tightly converged geometry optimization, a threshold value of 5 × 10^−3^ eV Å^−1^ was used for the magnitude of forces acting on nuclei. Scalar relativistic corrections were employed, using the atomic zeroth order regular approximation (ZORA).^[^ ^39^ ^]^ 16‐ and 28‐atom unit cells were used for HfO_2_ and Hf_3_N_4_, respectively.

The procedure for computing defect formation energies has the following main features.^[^ ^22^, ^43^, ^44^ ^]^ and the main features are described here. The expression for the formation energy was

(1) $$E_{form}=E_{defect}\;-E_{pristine}+\sum_{i}n_{i}\mu_{i}$$

where *n*_*i*_ was the number of atoms added or removed to form the defect, and *µ*_*i*_ was the chemical potential. Since only neutral defects were considered, there was no need to account for variations in the Fermi energy, or to apply electrostatic corrections to account for image charge interactions.

The chemical potentials were not constant, instead varying between certain limits defined by the thermodynamics of the system. O‐ and N‐rich limits were defined by:

(2) $$\mu_{N}\leq \frac{\mu_{N_{2}}}{2},\;\mu_{O}\leq \frac{\mu_{O_{2}}}{2}$$

The enthalpies of formation of stoichiometric HfO_2_ and Hf_3_N_4_ place additional constraints on the chemical potentials, defining the O‐ and N‐poor limits, respectively:

(3) $${\Delta E}_{f}^{HfO_{2}}=\mu_{HfO_{2}}-\mu_{Hf}-2\mu_{O},{\Delta E}_{f}^{HfO_{2}}\leq 0$$

(4) $${\Delta E}_{f}^{Hf_{3}N_{4}}=\mu_{Hf_{3}N_{4}}\;-3\mu_{Hf}-4\mu_{N},{\Delta E}_{f}^{Hf_{3}N_{4}}\leq 0$$

For results reported in the main text, the O‐poor limit was used, in line with described experimental conditions (only residual oxygen was present in the ALD chamber). Similar synthetic procedures for HfO_2_ lead to high concentrations of oxygen vacancies^[^ ^37^ ^]^ and sub‐stoichiometric Hf:O ratios of 1.91 or less.^[^ ^45^ ^]^ As for nitrogen, an average chemical potential between the N‐rich and N‐poor limits was chosen. A full summary of all possible limits is included in Tables S1–S4 (Supporting information).

Estimates of defect concentrations can be obtained using an Arrhenius‐like expression, assuming a deposition temperature of 423 K (similar to the experimental ALD temperature):

(5) $$n=\frac{n_{0}}{1+e^{\frac{E_{form}}{kT}}}$$

Using the methods outlined previously, 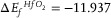 eV/unit cell, in line with the experimental enthalpy of formation of − 11.864 eV/unit cell were obtained.^[^ ^46^ ^]^

## Conflict of Interest

MIT has filed a patent application for the two‐part interlayer that names N.N., C.F.P., and M.A.B. as inventors (Application No.: 18/689,764).

## Supporting information



Supporting Information

## Data Availability

The data that support the findings of this study are available from the corresponding author upon reasonable request.
