# Supplementary material for: Triplet Exciton Sensitization of Silicon Mediated by Defect States in Hafnium Oxynitride
Source: Adv Mater. 2024 Dec 23;37(7):2415110. doi: 10.1002/adma.202415110 (PMC11837895; doi:10.1002/adma.202415110)
Supplement: Supplementary file 1 — Supporting Information [file ADMA-37-2415110-s001.docx]

Supporting Information

**Triplet Exciton Sensitization of Silicon Mediated by Defect States in Hafnium Oxynitride**

Narumi Nagaya*, Alexandra Alexiu, Collin F. Perkinson, Oliver M. Nix, Dooyong Koh, Moungi G. Bawendi, William A. Tisdale, Troy Van Voorhis, Marc A. Baldo

**X-ray photoelectron spectroscopy of hafnium oxynitride and hafnium oxide films**


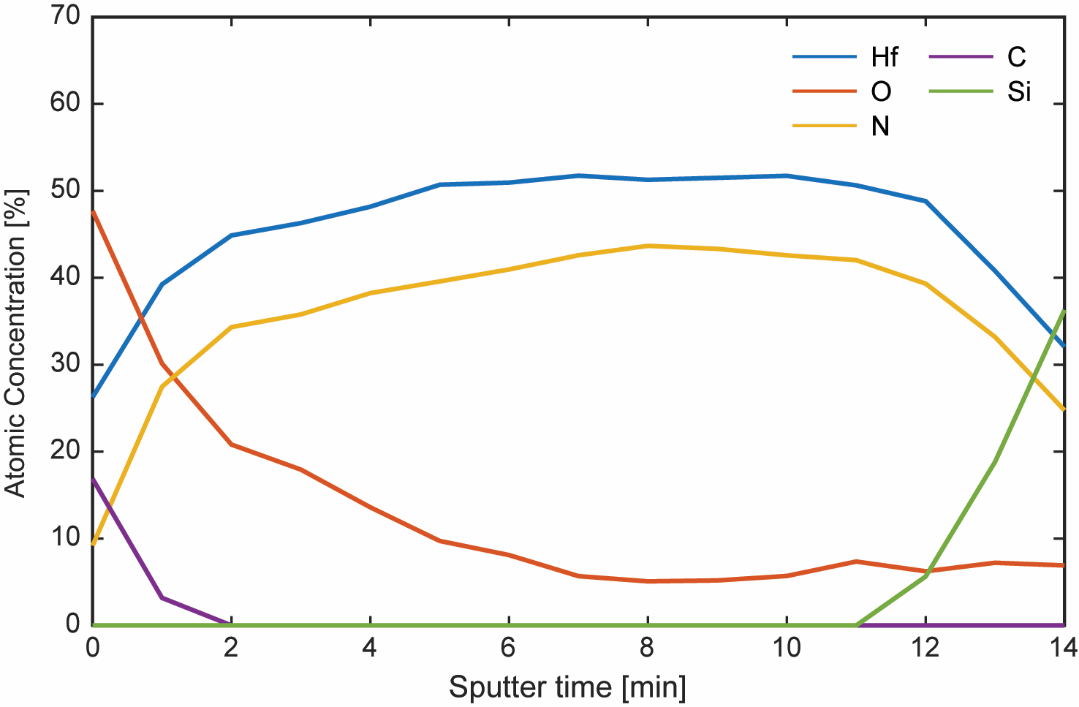


**Figure S1.** Compositional depth-profiles obtained from X-ray photoelectron spectroscopy measurements on the HfO_x_N_y_ film deposited using tetrakis(dimethylamino)hafnium (TDMAH) and NH_3_ precursors. C_60_ ions were used to sputter the surface, and the photoelectron peak areas for C1s, N1s, O1s, Si2p, Hf4f were measured in 1-minute sputtering intervals.


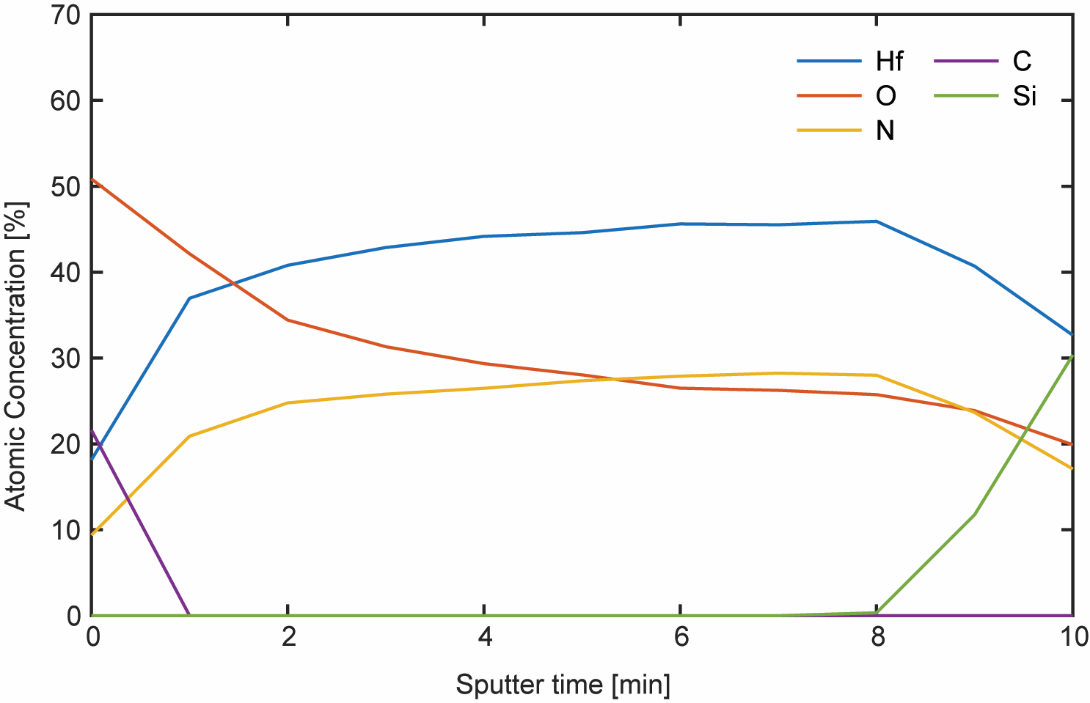


**Figure S2.** Compositional depth-profiles obtained from X-ray photoelectron spectroscopy measurements on the HfO_x_N_y_ film deposited using tetrakis(ethylmethylamino)hafnium (TEMAH) and NH_3_ precursors. C_60_ ions were used to sputter the surface, and the photoelectron peak areas for C1s, N1s, O1s, Si2p, Hf4f were measured in 1-minute sputtering intervals.


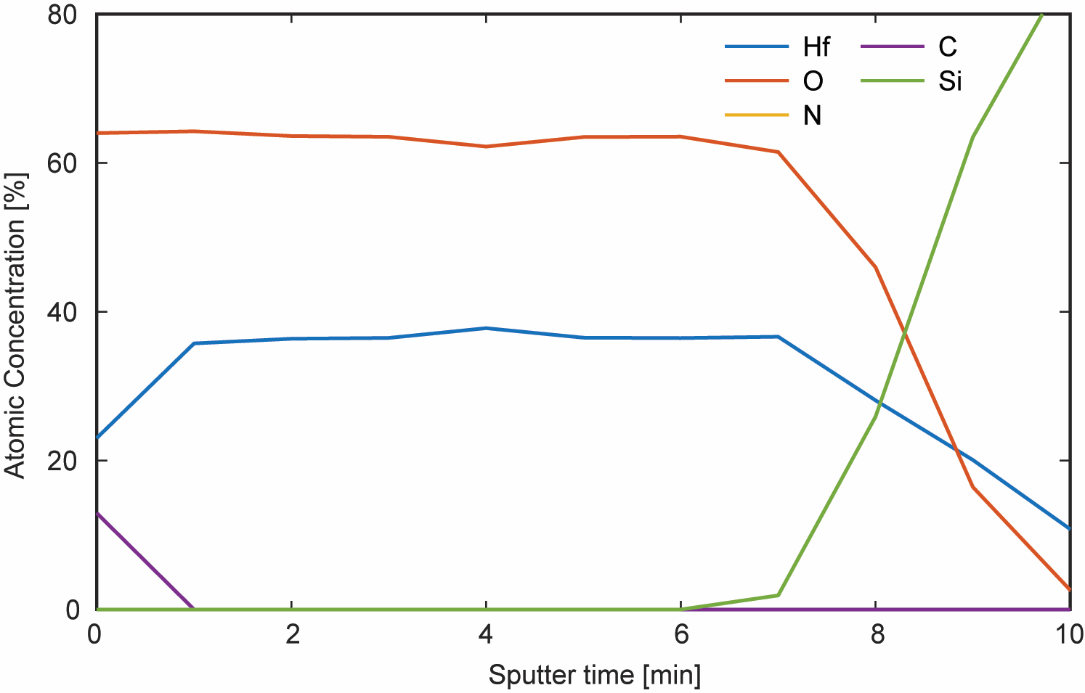


**Figure S3.** Compositional depth-profiles obtained from X-ray photoelectron spectroscopy measurements on the HfO_x_ film deposited using tetrakis(ethylmethylamino)hafnium (TEMAH) and H_2_O precursors. C_60_ ions were used to sputter the surface, and the photoelectron peak areas for C1s, N1s, O1s, Si2p, Hf4f were measured in 1-minute sputtering intervals.

**Photoluminescence spectrum of neat tetracene film**

**
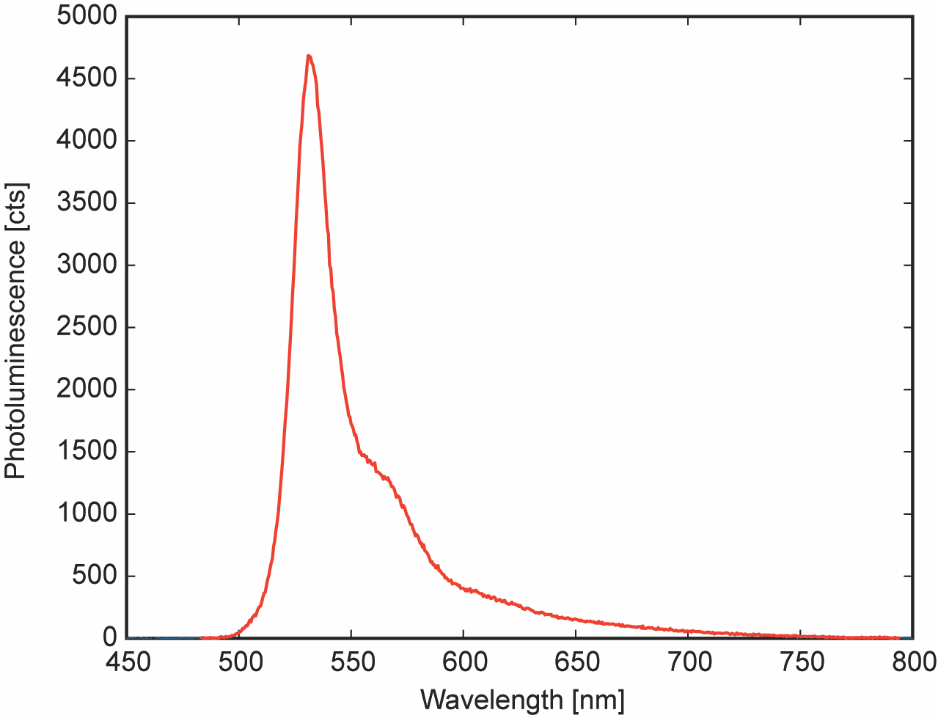
**

**Figure S4.** Photoluminescence spectrum of a 30 nm neat tetracene film. The sample was excited using a 405 nm laser source to show the full spectrum with no overlap of the laser signal. In the experiment of the main text, we use both a 532 nm notch filter and a 550 nm longpass filter to block the laser overlap, capturing the shoulder of the tetracene photoluminescence. For the tetracene on silicon samples, we use both a 900 nm longpass filter and a 1000 nm longpass filter to block the laser signal as well as the tetracene photoluminescence to capture the silicon photoluminescence only.

**Additional computational investigations of hafnium oxynitride and hafnium oxide films**

**
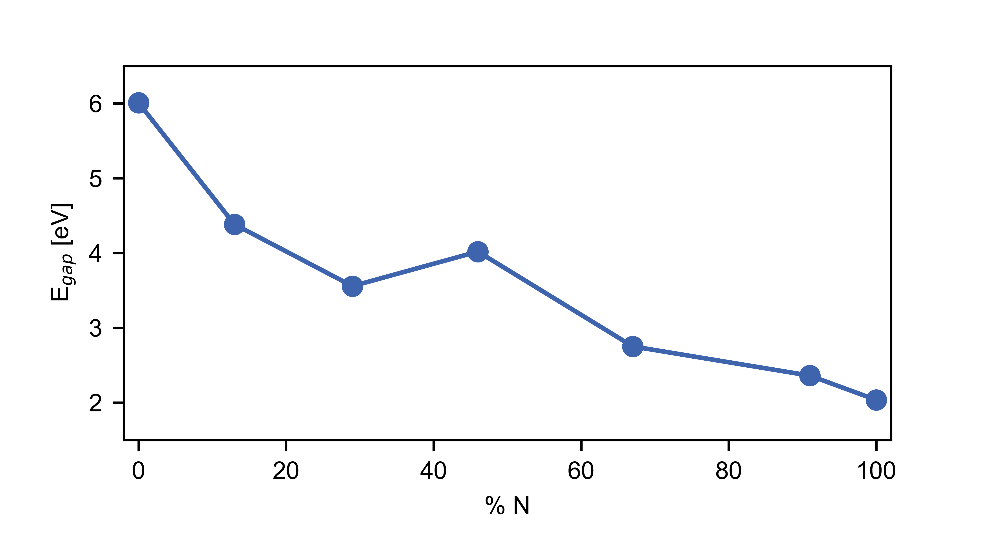
**

**Figure S5.** Band gap variation with N composition.

**
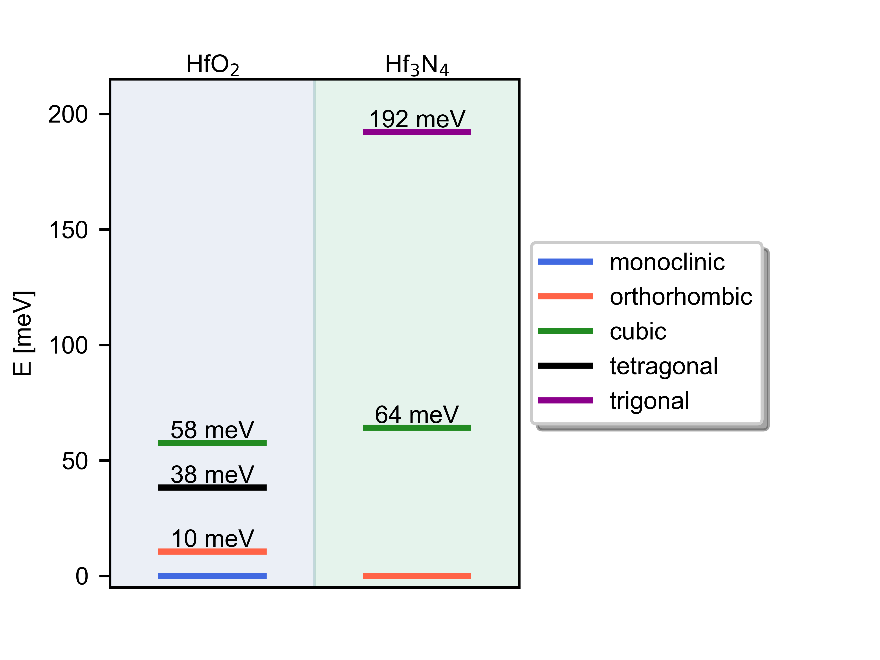
**

**Figure S6.** Relative energies of HfO_2_ and Hf_3_N_4_ phases.

**
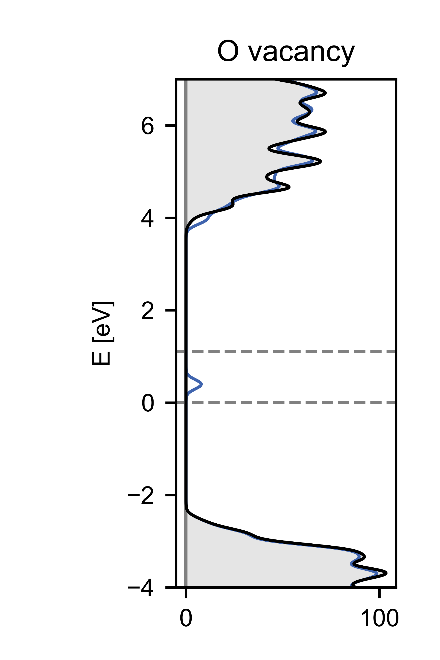

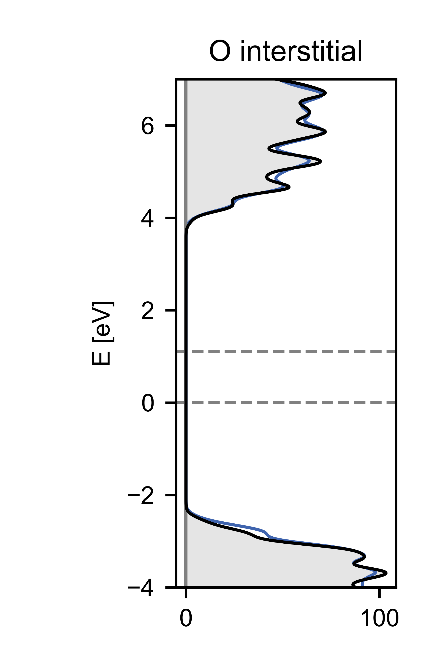

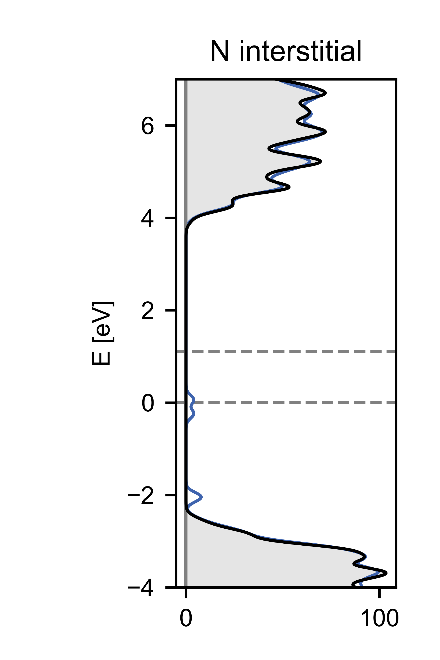
**

**Figure S7.** Density of states plots for O vacancy, O interstitial, and N interstitial defects in monoclinic HfO_2_. The DOS of the pristine unit cell (grey shaded region) is compared with the defective DOS (colored). The Si valence band maximum (VBM) and conduction band minimum (CBM) are marked by dotted grey lines. The energy axis is shifted such that the Si VBM is at 0 energy.

**
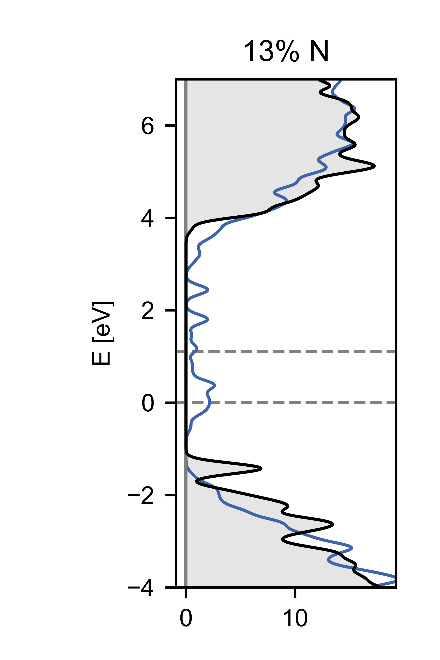

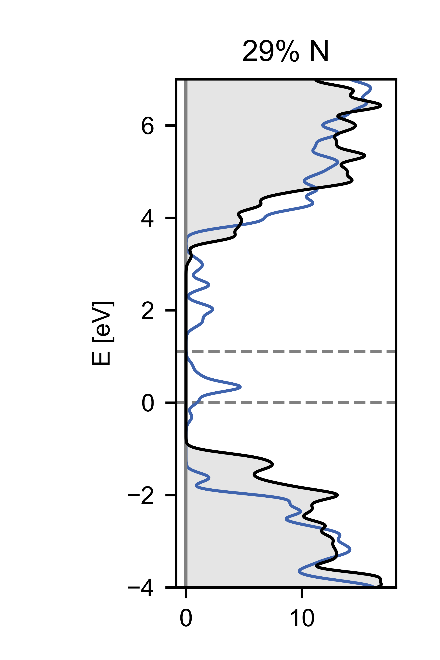

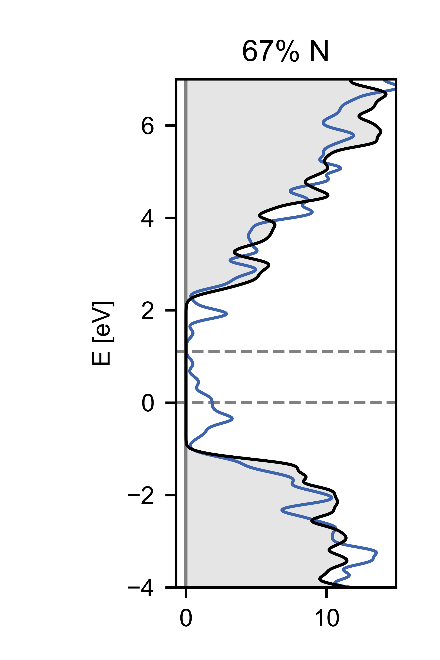

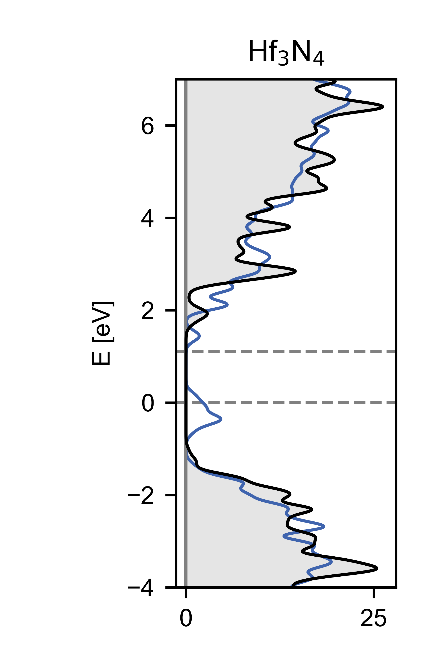
**

**Figure S8.** Density of states plots for N vacancy defects in a range of HfO_x_N_y_ compositions.

**
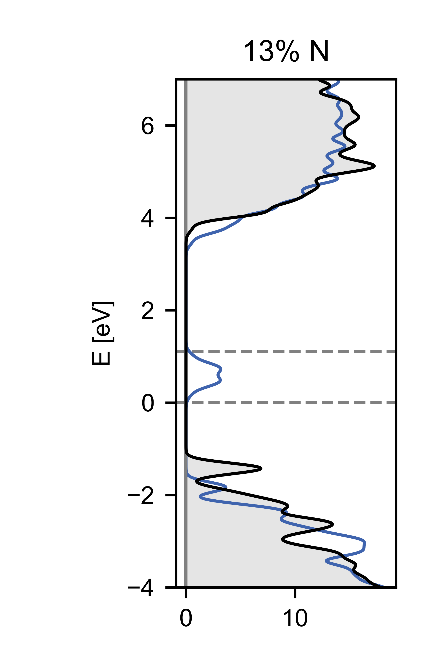

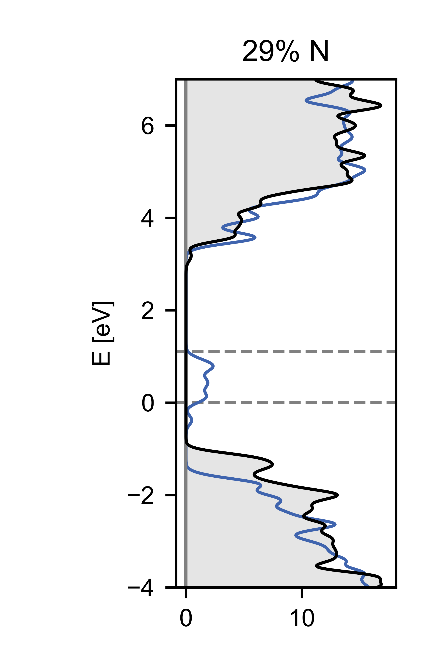

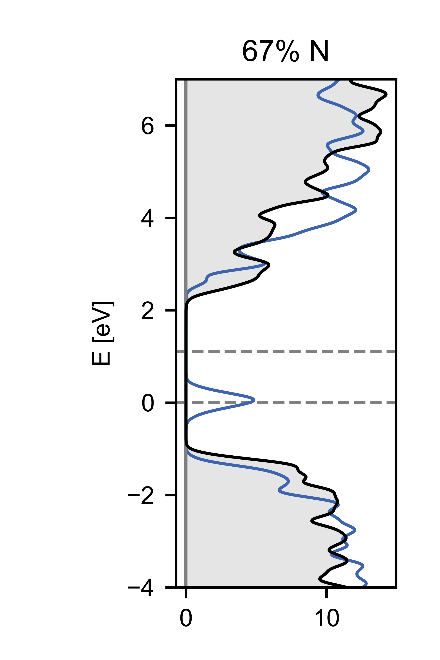
**

**Figure S9.** Density of states plots for O vacancy defects in a range of HfO_x_N_y_ compositions.

**
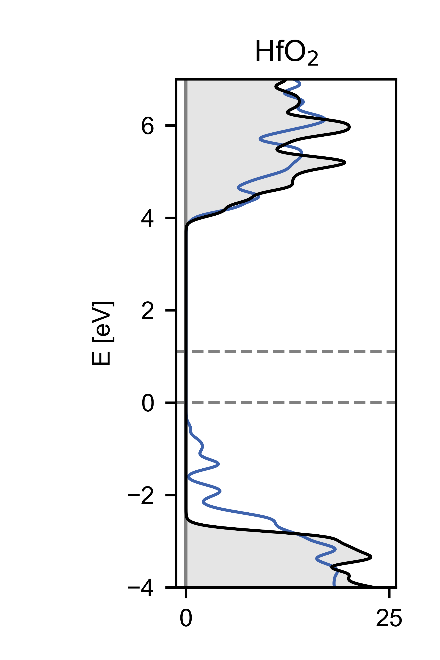

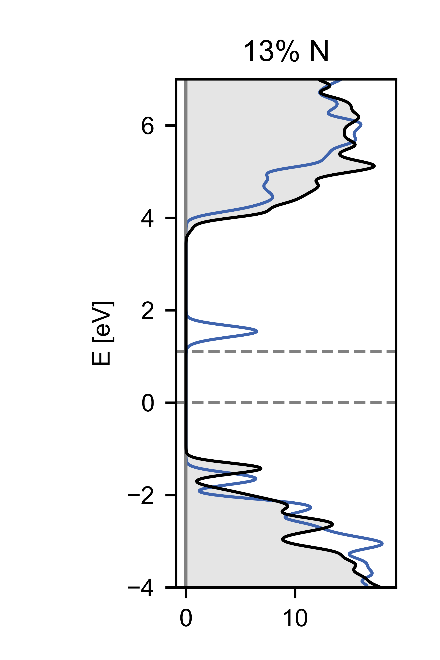

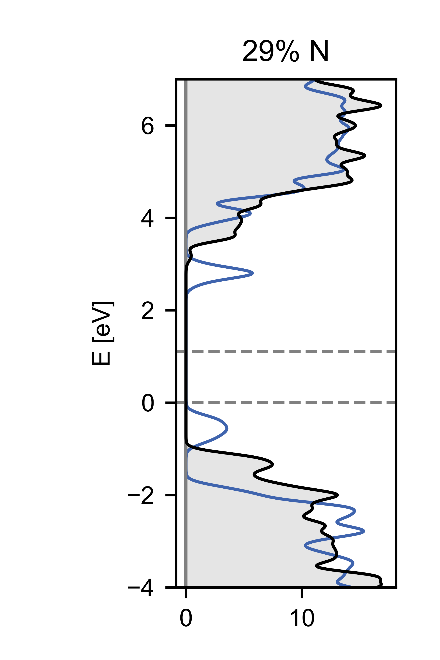

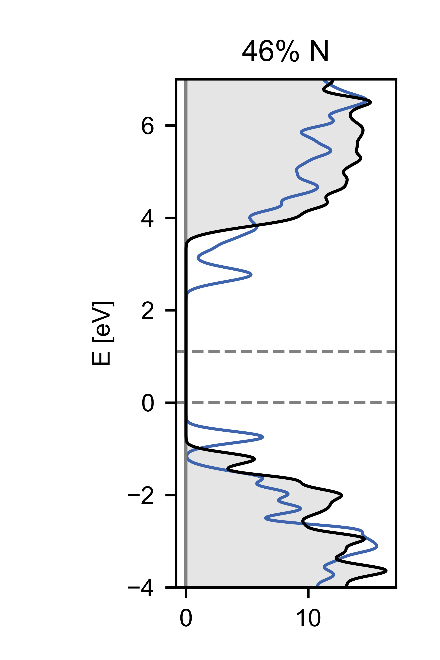

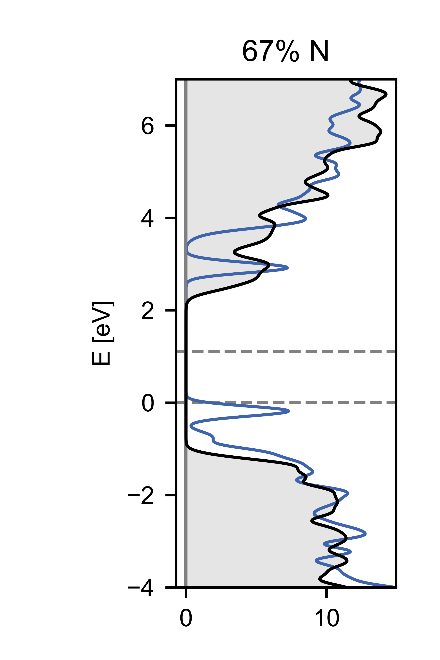

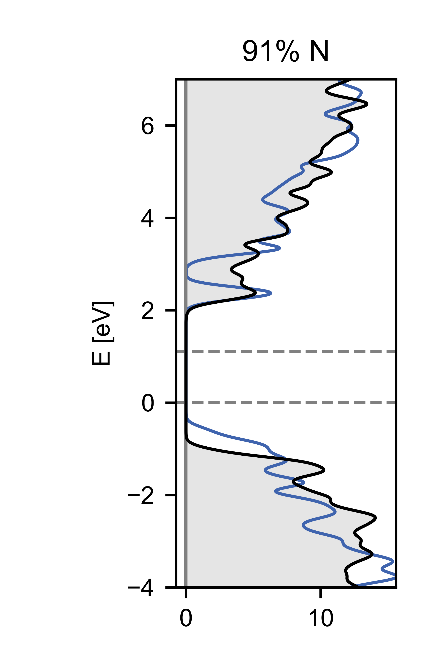

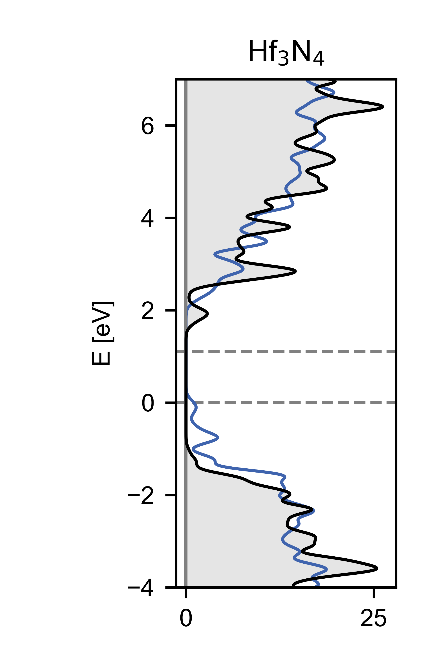
**

**Figure S10.** Density of states plots for Hf vacancy defects in a range of HfO_x_N_y_ compositions.

**
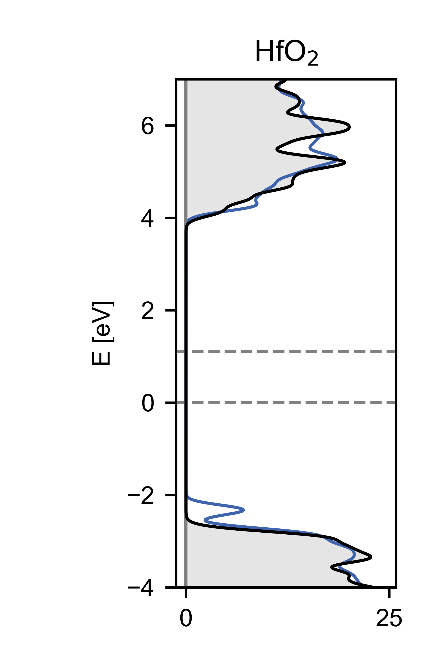

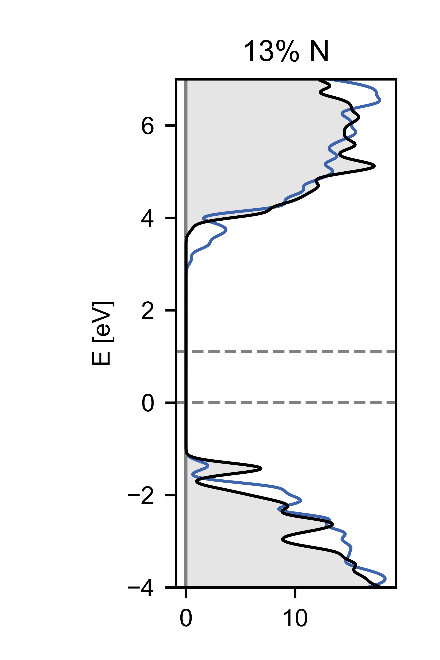

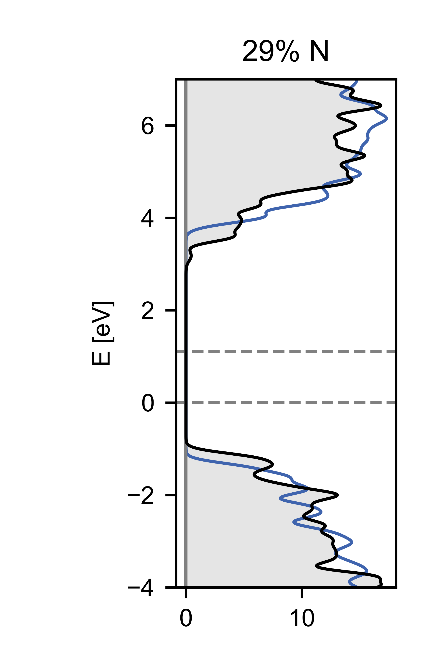

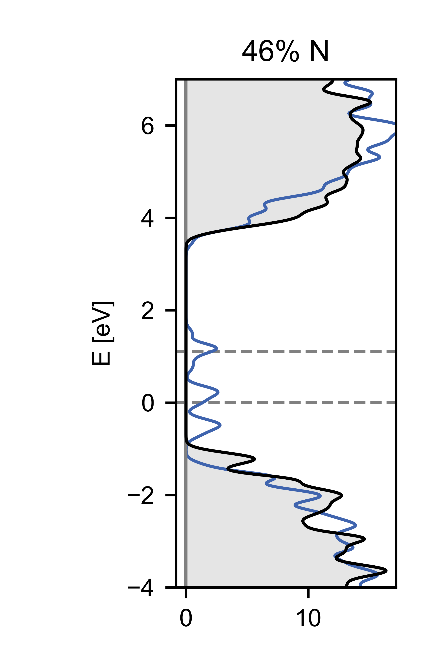

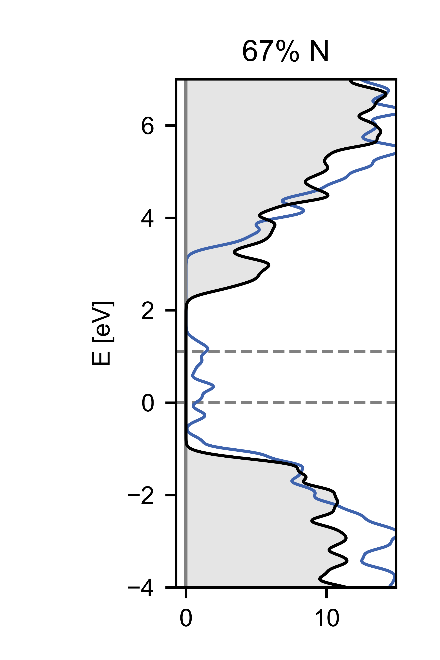

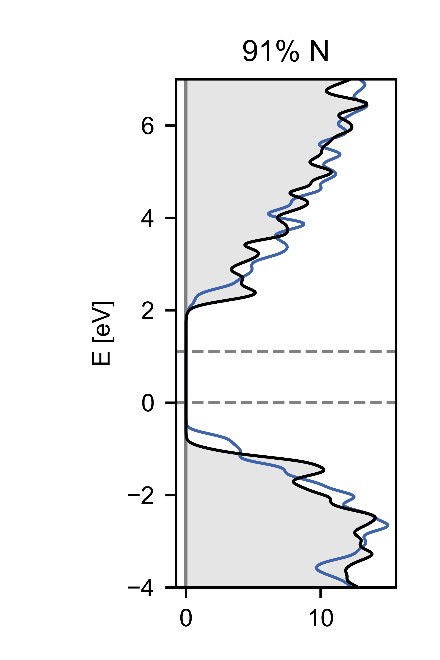

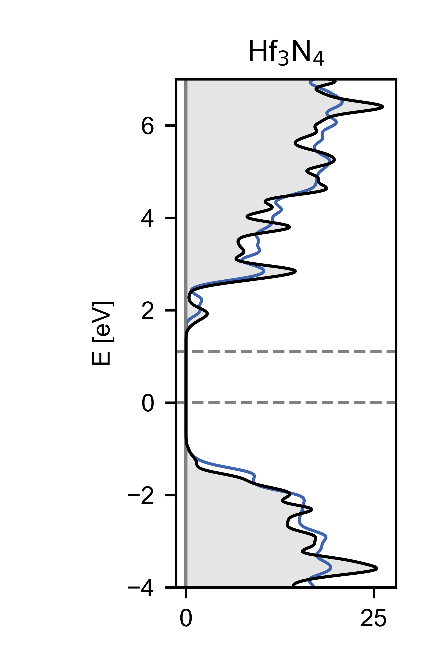
**

**Figure S11.** Density of states plots for O interstitial defects in a range of HfO_x_N_y_ compositions.

**
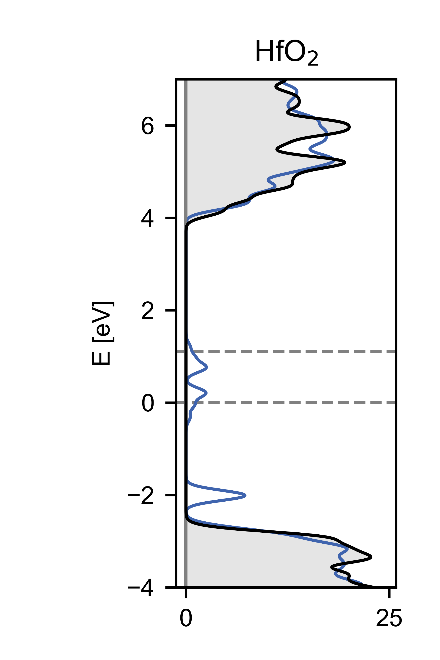

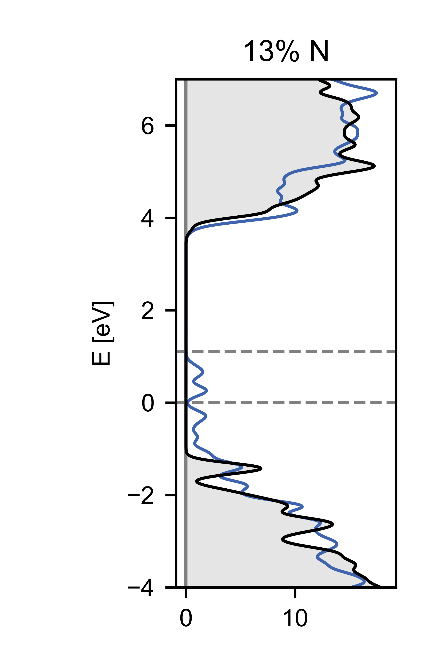

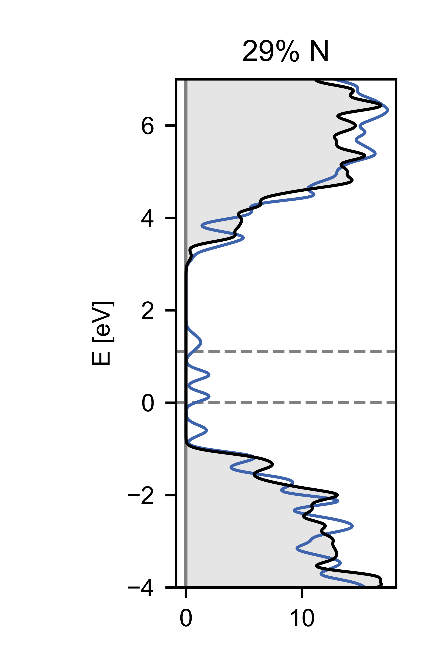

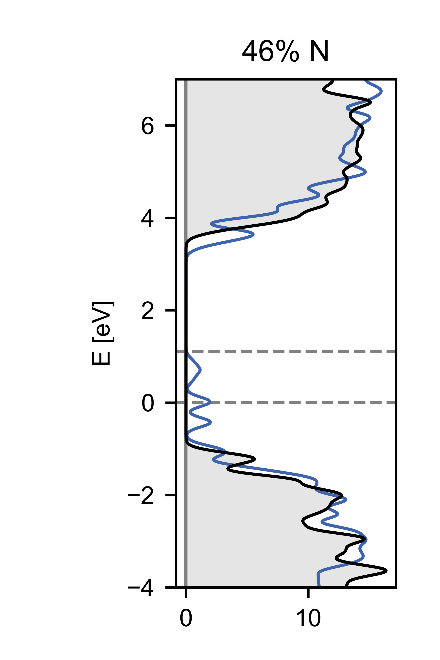

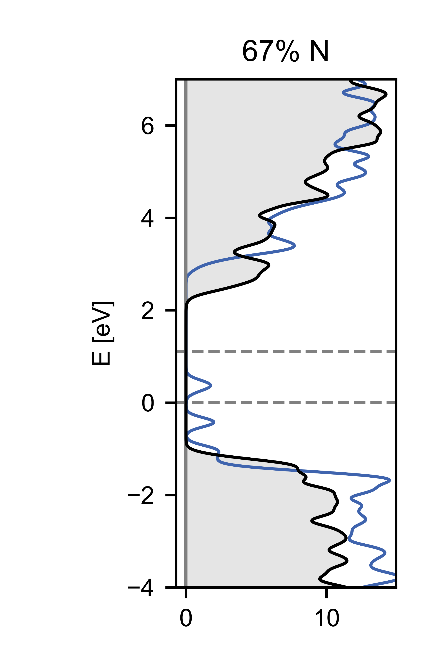

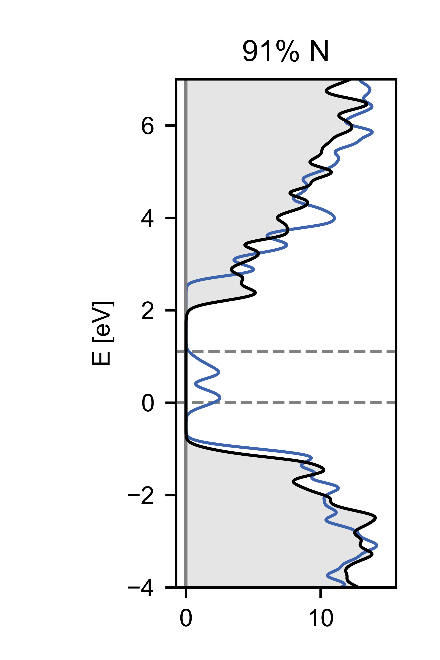

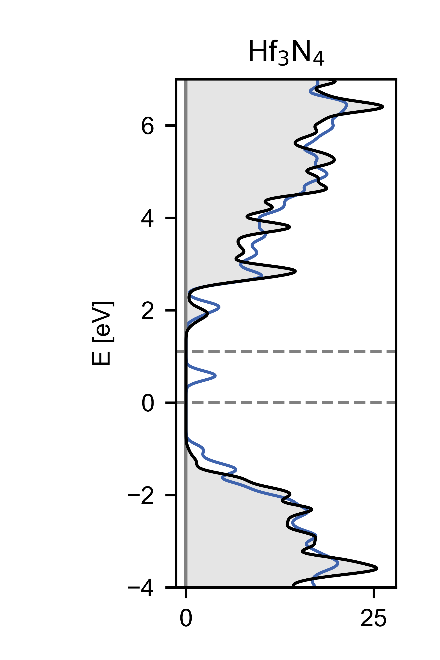
**

**Figure S12.** Density of states plots for N interstitial defects in a range of HfO_x_N_y_ compositions.

**
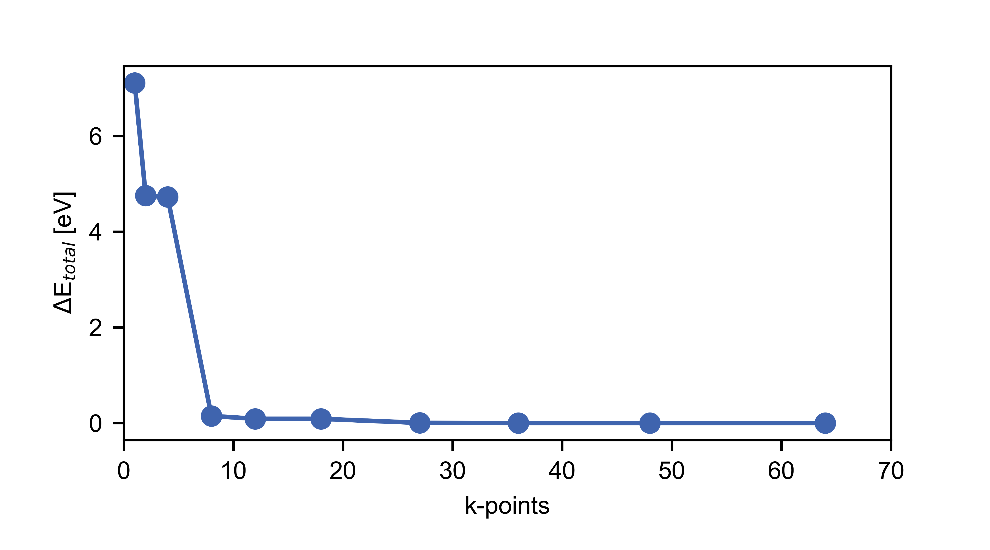
**

**Figure S13.** Convergence of total energy for the orthorhombic HfO_2_ unit cell with increasing k-points.

**
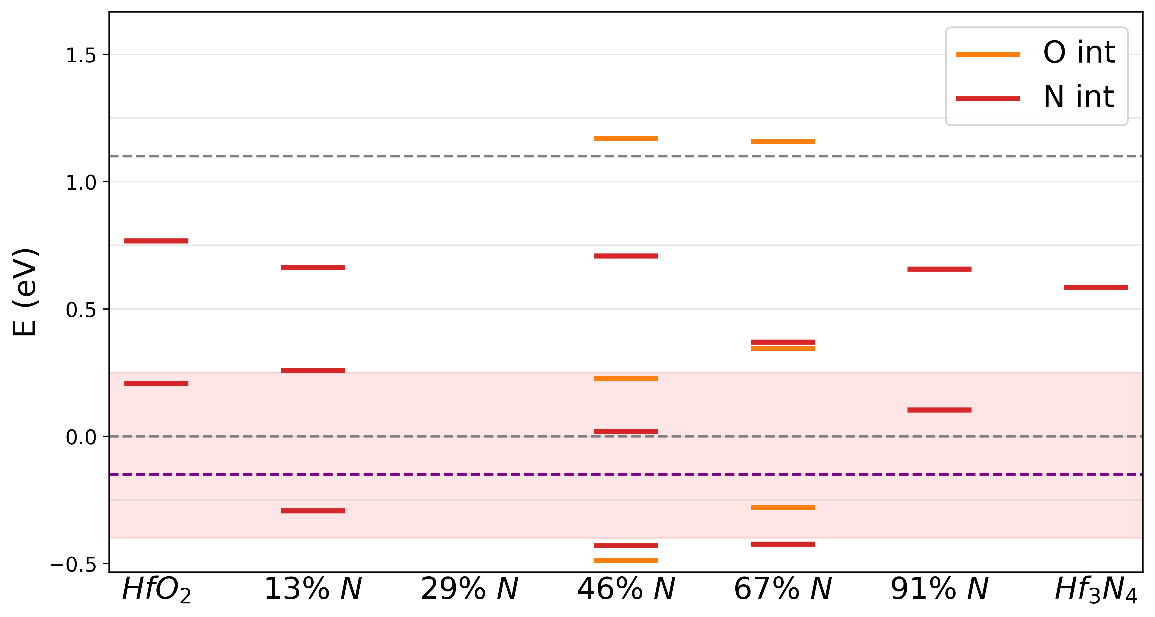
**

**Figure S14.** Summary of defect energy levels caused by O interstitials (orange) and N interstitials (red). The Si valence band maximum (VBM) and conduction band minimum (CBM) are marked with dotted grey lines, and the dotted purple line represents the minimum energy a defect state could have such that $\boldsymbol{E}_{\boldsymbol{\pm}}\boldsymbol{\leq}\boldsymbol{E}_{\boldsymbol{T}\boldsymbol{,}\boldsymbol{Tc}}$. The red shaded box marks the energy region for potential hole traps. The energy axis is shifted such that the Si VBM is at 0 energy.


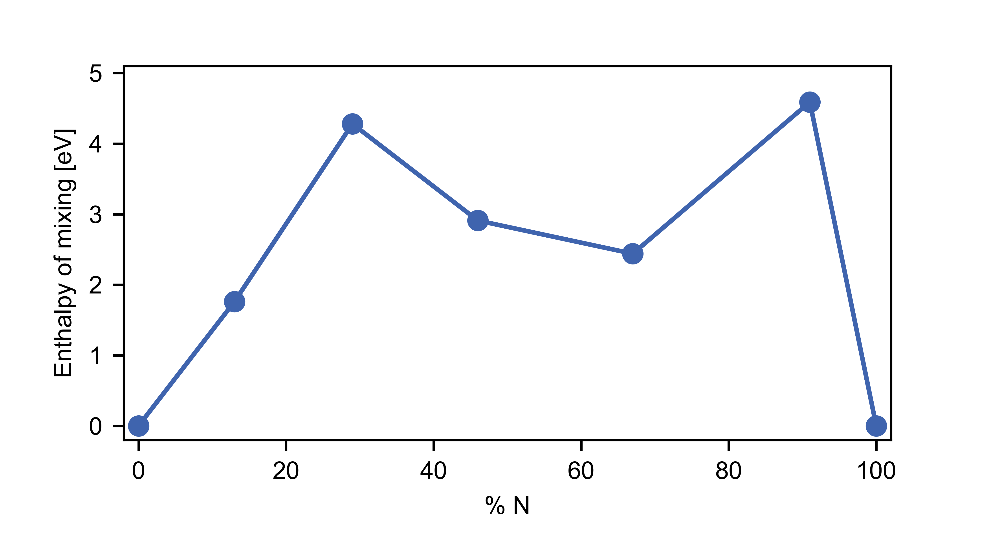


**Figure S15.** Enthalpy of mixing variation with increasing N composition, with respect to the pure end-members HfO_2_ and Hf_3_N_4_.

**Table 1.** Formation energies for N interstitial defects.

| Defect formation energy  [eV] | HfO_2_ | 13% N | 29% N | 46% N | 67% N | 91% N | Hf_3_N_4_ |
| --- | --- | --- | --- | --- | --- | --- | --- |
| N rich | 5.64 | 4.29 | 6.06 | 4.34 | 5.91 | 2.42 | 3.35 |
| N intermediate | 7.55 | 6.20 | 7.97 | 6.25 | 7.82 | 4.33 | 5.26 |
| N poor | 9.45 | 8.11 | 9.87 | 8.16 | 9.73 | 6.24 | 7.17 |

**Table 2.** Formation energies for N vacancy defects.

| Defect formation energy  [eV] | 13% N | 29% N | 46% N | 67% N | 91% N | Hf_3_N_4_ |
| --- | --- | --- | --- | --- | --- | --- |
| N rich | 3.60 | 2.37 | 2.62 | 2.44 | 0.90 | 3.29 |
| N intermediate | 1.69 | 0.47 | 0.71 | 0.53 | -1.01 | 1.39 |
| N poor | -0.22 | -1.44 | -1.20 | -1.38 | -2.92 | -0.53 |

**Table 3.** Formation energies for O interstitial defects.

| Defect formation energy  [eV] | HfO_2_ | 13% N | 29% N | 46% N | 67% N | 91% N | Hf_3_N_4_ |
| --- | --- | --- | --- | --- | --- | --- | --- |
| O rich | 2.11 | 0.67 | -0.07 | -0.95 | 1.47 | -1.48 | 0.41 |
| O intermediate | 5.00 | 3.65 | 2.92 | 2.03 | 4.45 | 1.51 | 3.39 |
| O poor | 7.89 | 6.63 | 5.90 | 5.02 | 7.43 | 4.49 | 6.37 |

**Table 4.** Formation energies for O vacancy defects.

| Defect formation energy  [eV] | HfO_2_ | 13% N | 29% N | 46% N | 67% N | 91% N |
| --- | --- | --- | --- | --- | --- | --- |
| O rich | 6.37 | 5.62 | 4.96 | 6.57 | 5.88 | 4.09 |
| O intermediate | 3.39 | 2.55 | 1.31 | 3.48 | 2.74 | 1.10 |
| O poor | 0.40 | -0.53 | -2.33 | 0.39 | -0.39 | -1.88 |

**Table 5.** Defect concentration, assuming O poor, N intermediate limits, and T = 423 K.

| Concentration [cm^-3^] | HfO_2_ | 13% N | 29% N | 46% N | 67% N | 91% N | Hf_3_N_4_ |
| --- | --- | --- | --- | --- | --- | --- | --- |
| O vacancy | 4.17 × 10^16^ | 3.75 × 10^21^ | 3.75 × 10^21^ | 6.46 × 10^16^ | 3.75 × 10^21^ | 3.75 × 10^21^ | N/A |
| N vacancy | N/A | 5.96 × 10^0^ | 7.05 × 10^15^ | 7.04 × 10^12^ | 1.21 × 10^15^ | 3.75 × 10^21^ | 2.74 × 10^4^ |
| O interstitial | 2.17 × 10^-76^ | 6.95 × 10^-61^ | 7.75 × 10^-52^ | 5.93 × 10^-41^ | 9.42 × 10^-71^ | 1.67 × 10^-34^ | 1.11 × 10^-57^ |
| N interstitial | 4.06 × 10^-72^ | 1.45 × 10^-55^ | 2.69 × 10^-77^ | 3.59 × 10^-56^ | 1.77 × 10^-75^ | 1.83 × 10^-32^ | 4.15 × 10^-34^ |
